# Unraveling mysteries associated with cat-scratch disease, bacillary angiomatosis, and related syndromes.

**DOI:** 10.3201/eid0101.950103

**Published:** 1995

**Authors:** R Regnery, J Tappero

**Affiliations:** National Center for Infectious Diseases, Centers for Disease Control and Prevention, Atlanta, GA 30333, USA. rur1@ciddvd1.em.cdc.gov

## Abstract

The search for the infectious agents responsible for cat-scratch disease, bacillary angiomatosis, and related syndromes has a long and often circuitous history. Recognition of the etiologic agents and a new understanding of the fundamental features of the epidemiology and natural history of modern day Bartonella (formerly Rochalimaea)-associated diseases culminate a multipartite story that combines clinical medicine, traditional microbiology, and novel technological approaches to solve a long-standing enigma.


### Unraveling Mysteries Associated with Cat-Scratch Disease, Bacillary Angiomatosis, and Related Syndromes

**Russell Regnery, Ph.D., and Jordan Tappero, M.D.**
National Center for Infectious Diseases,
Centers for Disease Control and Prevention, Atlanta, Georgia, USA

*The search for the infectious agents responsible for cat-scratch disease, bacillary angiomatosis, and related syndromes has a long and often circuitous history. Recognition of the etiologic agents and a new understanding of the fundamental features of the epidemiology and natural history of modern day* Bartonella *(formerly* Rochalimaea*)-associated diseases culminate a multipartite story that combines clinical medicine, traditional microbiology, and novel technological approaches to solve a long-standing enigma.*

The quest for the etiologic agent of cat-scratch disease (CSD) has frequently been described as a mystery (1,2). Indeed, the search has many qualities of a mystery novel; the pursuit has spanned several decades and recently taken several unexpected turns. During this period of important discovery, major microbial suspects have undergone name changes, novel microbial culprits have been introduced, new groups of affected patients have been recognized, and yet significant questions remain to be answered. Scientific and medical interest has been high; approximately 900 publications have dealt with CSD since the first good clinical description of the disease in 1950 (3).

### Clinical Features of CSD

Throughout the life of this evolving mystery, the clinical descriptions of "classical" CSD have remained remarkably consistent (Dalton MJ, et al. *Rochalimaea* antibody; a new era in the diagnosis of cat-scratch disease, submitted for publication; 4-6). CSD is typically a benign and self-limited illness lasting 6 to 12 weeks in the absence of antibiotic therapy. Regional lymphadenopathy (axillary, head and neck, inguinal) is the predominant clinical feature of CSD; affected nodes are often tender and occasionally suppurate (4-7). Between 25% and 60% of patients report a primary cutaneous inoculation lesion (0.5- to 1-cm papule or pustule) at the site of a cat scratch or bite (5,7). The skin lesions typically develop 3 to 10 days after injury and precede the onset of lymphadenopathy by 1 to 2 weeks. Low-grade fever and malaise accompany lymphadenopathy in up to 50% of patients; headache, anorexia, weight loss, nausea and vomiting, sore throat, and splenomegaly may develop. In addition, short-lived, non-specific maculopapular eruptions, erythema nodosum, figurate erythemas, and thrombocytopenic purpura have been observed (8).

Unusual manifestations of CSD, which occur in up to 14% of patients, include Perinaud's oculoglandular syndrome (6%), encephalopathy (2%), hepatic granulomas (0.3%), osteomyelitis (0.3%), and pulmonary disease (0.2%) (4,5,8). In general, these complications resolve without sequelae. Perinaud's oculoglandular syndrome is manifested by conjunctival granuloma, periauricular lymphadenopathy, and nonsuppurative conjunctivitis. Encephalopathy, manifested as fever and coma that progress to convulsions, may last for days to weeks; cerebrospinal fluid is unremarkable. Optic neuritis with transient blindness may also occur.

For many years, CSD has been clinically diagnosed when three of the following four criteria are met in a patient: 1) history of traumatic cat contact; 2) positive skin-test response to CSD skin-test antigen; 3) characteristic lymph node lesions; and 4) negative laboratory investigation for unexplained lymphadenopathy (8). Although biopsy confirmation of CSD has been rarely required (especially in lieu of a reliable serologic test—see below), a constant pathologic hallmark of CSD-affected tissues has been granuloma formation. With hematoxylin and eosin stains, the primary inoculation lesion of CSD reveals small areas of frank necrosis surrounded by concentric layers of histiocytes, lymphocytes, and nucleated giant cells (9). Affected lymph nodes are characterized by necrotizing granulomas ringed by lymphocytic infiltrates and multinucleated giant cells.

Address for correspondence: Russell Regnery, Centers for Disease Control and Prevention, 1600 Clifton Road, MS G13, Atlanta, GA 30333, USA; fax 404-639-1058; e-mail rur1@ciddvd1.em.cdc.gov.

### Enter *Afipia felis*

During the past 44 years, a variety of microbial agents, including herpes viruses and bacteria of the genera *Chlamydia* and *Pasteurella*, have been suspected as the causes of CSD (3). A major chapter of the CSD saga unfolded with the 1988 announcement by the Armed Forces Institute of Pathology that a bacterial agent had been visualized within CSD-involved lymph nodes by using the Warthin-Starry silver stain (10), and a novel bacterial agent had been isolated from a CSD patient's lymph node (11). By 1992, this agent was characterized fully, given the name *Afipia felis* (*Afipia* being a latinized acronym for the source of the original isolate, the Armed Forces Institute of Pathology, and *felis* referring to the presumed vertebrate vector of the human infection), and proclaimed the agent of CSD (12).

Although *A. felis* was declared the putative CSD bacillus, evidence of convincing patient humoral or cellular immune responses to laboratory cultured *A. felis* antigen was not forthcoming. Despite numerous attempts, other laboratories were unable to recover additional isolates of *A. felis* from CSD patients. In addition, although the majority of patients with CSD reported exposure to a cat(s), no clear link between cats and *A. felis* was demonstrated.

### Enter New Syndromes

The story of CSD took a significantly divergent path with the recognition that opportunistic infections were an important consideration for patients infected with human immunodeficiency virus (HIV). Bacillary angiomatosis (BA), a newly recognized disease characterized by cutaneous and subcutaneous vascular lesions containing bacillary organisms visualized by Warthin-Starry silver staining, was described predominantly among HIV-infected patients; however, bacterial isolates were not made or identified (13-15). Over the ensuing decade, the clinical spectrum of BA was expanded to include patients with single or multiple vascular lesions affecting virtually every organ system, including lymph node, bone, brain, liver (peliosis hepatis), and spleen (14-17). Independently, an unidentified gram-negative pathogen was isolated predominantly from HIV-infected patients with fever and bacteremia, however, these patients lacked cutaneous or parenchymal vascular lesions and were not recognized as BA patients (18).

Because silver staining and electron microscopy of both BA and CSD tissue sections revealed bacillary organisms indistinguishable from one another, several authors suggested that BA might represent disseminated CSD in the immunocompromised host (17,19-21). In addition, several anecdotal reports of BA described a history of cat contact preceding the onset of disease (22).

Ultimately, the relationships between possible environmental exposures and BA or CSD were systematically investigated. The first case-control study conducted among patients with BA found traumatic contact with a cat (bite or scratch) to be significantly associated with BA disease (22). BA patients were also more likely than controls to have a household kitten (a cat <1 year of age). A subsequent case-control study of CSD patients found that these patients were more likely than controls to have traumatic contact with a cat, to own at least one kitten, and to have kittens with fleas (7).

Despite the similarities in histochemical staining properties and epidemiology, serious reservations remained concerning a possible link between the causative agents of CSD and BA. The pathologic features of classical CSD (granuloma) and BA (proliferative vascular lesions without granuloma) were distinctly different, and the two diseases seemed to respond differently to antibiotic therapy. Although antimicrobial therapy for BA and CSD have not been systematically evaluated, the majority of BA patients evaluated responded quickly to single-agent therapy with either erythromycin or doxycycline (14,23), whereas the symptoms and signs of patients with CSD failed to show consistent rapid resolution following antibiotic therapy (5). In addition, clinicians' first choices of antibiotics for treating BA and CSD vary (5,6,14,23).

### Enter *Rochalimaea henselae*

A breakthrough occurred when a novel approach was used to identify possible prokaryotic ribosomal DNA extracted from BA skin lesions. When prokaryotic ribosomal gene DNA extracted from BA lesions and amplified by polymerase chain reaction (PCR) was compared with sequenced ribosomal genes from other organisms, it become apparent that the agent associated with BA in this study was related to, but not necessarily identical to, the agent of trench fever, *Rochalimaea quintana* (24).

At nearly the same time in Oklahoma, *Rochalimaea*-like organisms were being isolated on blood agar from bacteremic patients (18). Independently in Houston, Texas, fastidious, slow-growing *Rochalimaea*-like isolates were recovered on several occasions from the blood of an HIV-infected patient with relapsing fever of unknown origin; like the isolates from the Oklahoma patients, the Houston isolate was recovered from a patient in the absence of BA or CSD lesions (25). The Houston isolate (Houston-1) was identified as the prototype isolate of a novel species of *Rochalimaea* by using traditional as well as genotypic methods, including ribosomal RNA gene analysis similar to that used to identify the nucleic acid found in BA patients' lesions (25). Almost simultaneously, the group from Oklahoma had come to a similar conclusion by using DNA relatedness data (26); most of their isolates also consisted of the novel species, *R. henselae.* The new species designation, first officially used to describe the Houston-1 isolate, was coined in recognition of the contribution of Diane Hensel, a microbiologist who had isolated several of the initial organisms in Oklahoma (18,25,26). Subsequently, Koehler et al. isolated bacilli directly from cutaneous lesions of persons with BA (27); surprisingly, either *R. henselae* or *R. quintana* was isolated from BA lesions from different HIV-infected patients.

At this juncture, *R. henselae* infections had been described predominantly among immunocompromised patients with either BA or fever with bacteremia. The availability of isolates made it possible to develop a test for serologic evidence of *Rochalimaea* infection and to refine PCR methods for identification of *Rochalimaea* organisms in tissues and other samples. These methods, together with new techniques for recovering *Rochalimaea* species isolates, were crucial to obtaining a more detailed account not only of BA but also of CSD.

### The Cat-scratch Connection: A Synthesis

A *Rochalimaea* genus-specific, indirect fluorescence antibody (IFA) test using irradiated whole cell antigen from the Houston-1 isolate of *R. henselae* was developed by the Centers for Disease Control and Prevention (CDC) to help identify risk factors for *Rochalimaea*-associated disease. Several blinded serum samples from both HIV-infected BA patients and HIV-infected controls residing in San Francisco were sent to CDC for serologic testing. High-titered antibodies were identified in serum samples from several of the BA patients (28). Similar high-titered antibodies were not detected for any of non-BA control patients with one exception; a serum sample from an HIV-infected patient with CSD also demonstrated strong serologic reactivity to *R. henselae* antigen.

Shortly thereafter, single sera collected from patients with suspected CSD to look for *A. felis* antibodies were evaluated with the new *R. henselae* serologic test; 36 (88%) of 41 sera were positive (29). None of the sera had significantly elevated titers to *A. felis* antigen. The same set of sera were coded and resubmitted along with sera taken from other well-characterized bacterial and viral diseases and tested again in a blinded manner. The IFA test accurately identified sera of case-patients with suspected CSD. In addition, 6 (6%) of 107 sera from ostensibly healthy persons, obtained from a commercial vendor, showed antibody by IFA testing (29). These serologic data were the first laboratory evidence suggesting that *R. henselae* was associated with CSD.

Data further substantiating the role of *R. henselae* in the etiology of CSD soon followed. The newly developed serologic test was used to help investigate a possible cluster of CSD cases in Connecticut; 38 (84%) of 45 suspected CSD cases had elevated *Rochalimaea* antibody titers, whereas 4 (3.6%) of 112 age-matched controls had detectable antibody titers (7). In another investigation, serum samples obtained from 600 prospectively evaluated patients with well-characterized CSD (i.e., persons with history of cat scratch, papule at site of inoculation, and enlarged regional lymph node) had a 95% correlation with positive *Rochalimaea* serology.

In 1993, *R. henselae* was isolated directly from the lymph nodes of two CSD patients and was identified by genotypic means; both patients had strong serologic responses to *Rochalimaea* antigen (30). Evidence of *R. henselae*-specific nucleic acid sequences were found in 21 (84%) of 25 CSD lymph node tissues submitted to CDC for evaluation (31).

Additional supporting evidence for a *Rochalimaea* as the cause of CSD came from archival sources. Skin-test antigen, used rather extensively in the past to help diagnose examples of CSD (4,8), consisted of pasteurized exudate collected from suppurative CSD lymph nodes. Among a cohort of CSD patients who were skin-test positive, 52 (93%) of 56 had positive IFA antibody titers to the defined *Rochalimaea* reagents (32). Furthermore, various lots of skin-test antigen were shown by PCR analysis to contain *Rochalimaea* nucleic acid sequences (33), and *R. henselae* sequences in particular (34). No *A. felis* DNA sequences could be detected by PCR. These data strongly indicated that microbiologically undefined skin-test reagents, which had been used for many years for the diagnosis and clinical characterization of CSD, were in fact *R. henselae* reagents.

Collectively, these data supported a *Rochalimaea* species etiology for both CSD and BA. Despite numerous attempts, recent efforts to implicate *A. felis* as a cause of either of these two clinical entities have repeatedly failed.

### *Felis domesticus*: A Reservoir for *Rochalimaea henselae*

In addition to epidemiologic data, serologic evidence also implicated domestic cats with *Rochalimaea*-associated disease. *Rochalimaea*-specific IFA antibodies were demonstrated in 6 (46%) of 13 pet cats not associated with human disease and among 39 (81%) of 48 cats living in households reporting human CSD in Connecticut (7). Microbiologic evidence for the domestic cat as a reservoir for *R. henselae* soon followed. *R. henselae* was isolated over a 3-week period from the blood of a single cat not linked to human illness (35). Investigations by Koehler et al. established the cat as a reservoir for

*R. henselae* infection (36). *R. henselae* was established as the cause of cutaneous BA in three or four patients with the disease. *R. henselae* was isolated from the blood of all seven asymptomatic pet cats with which these four BA patients had prolonged contact. The prevalence of infection among cats in the greater San Francisco Bay region was also studied; 25 (41%) of 61 pet or impounded cats had asymptomatic *R. henselae* bacteremia (36). *R. henselae* was also detected by both direct culture and PCR from several cat fleas combed from these bacteremic cats (36).

The human body louse (*Pediculus humanus)* was established as a vector for human-to-human trench fever *R. quintana* transmission during the First World War (37). Likewise, *B. bacilliformis*, a closely related organism (see below) found in the mountains of South America, can be transmitted by another arthropod, the *Phlebotomus* sand fly (38). The observation that related microbes are vectored between humans by arthropods adds credence to the proposed role of arthropod vectors of CSD. Despite several suggestions that fleas or possibly ticks (7,36,39) are associated with *R. henselae* disease, no experimental data exist to clearly demonstrate that arthropods act as direct vectors.

### Changes in Nomenclature: *Rochalimaea* becomes *Bartonella*

Genotypic evaluation of members of the genus *Rochalimaea* has led to the conclusion that members of the genus are closely related to *Bartonella bacilliformis*, the agent of Carrión disease, Oroya fever, and verruga peruana (40). Because of historical precedence, the genus designation *Bartonella* is now applied to all species of the old genus *Rochalimaea* and replaces the *Rochalimaea* designation (species names remain unchanged).

Physicians and researchers need to exercise care in using the term "bartonellosis." This term has classically been used to describe the frequently fatal syndromes caused by *B. bacilliformis*. To date, *B. bacilliformis* and its associated syndrome (bartonellosis) have been identified exclusively in South America (38,41).

### Remaining Questions for Ongoing and Future Research

Although *B. henselae* is now regarded as the etiologic agent of CSD, as well as a cause of BA, endocarditis (42), and fever with bacteremia, many questions remain unanswered. For example, why did it take so long to isolate and identify *B. henselae*? Part of the answer probably stems from the requirements necessary for growth in vitro, including enriched, non-selective blood agar incubated over a prolonged period in a $CO_2$ atmosphere. Most hospital laboratories discard their bacteriological plates before primary isolates of *B. henselae* would be expected to appear (9-40 days). Extreme sensitivity to a wide variety of antibiotics, at least in vitro, suggests that residual levels of antibiotics in patients' blood or other tissues (such as lymph node biopsy) might inhibit *Bartonella* growth during primary isolation attempts in vitro. Selective medium has yet to be developed. Novel genotypic methods were crucial for identification of *B. henselae*; thus, isolates may well have been made in the past but remained unidentified.

As mentioned above, it has become apparent that in addition to *B. henselae, B. quintana* can also be another significant cause of BA disease, at least among immunocompromised patients in San Francisco (27). Another focus of *B. quintana* infections ("urban trench fever") has been identified among homeless alcoholics in Seattle (43,44). How common are *B. quintana* infections; are they louseborne and vectored strictly between humans, as was believed during World War I (37)? *B. quintana*-associated disease has no known link with an alternative vertebrate vector (such as cats).

*Bartonella elizabethae* is known only from a single isolate from a man surviving endocarditis following aortic valve-replacement surgery (45). Is there further public health significance to this organism? What additional *Bartonella* species have yet to be identified and what diseases may they cause?

Members of the genus *Bartonella* are exquisitely sensitive to antibiotics in vitro (30,46). Why then do CSD patients not respond as rapidly and consistently to antibiotic therapy as BA patients do? One hypothesis is that immunocompetent patients somehow sequester infectious organisms beyond the reach of antibiotics, whereas immunocompromised patients do not. An alternative hypothesis regarding differential antibiotic responsiveness recognizes that many of the signs of CSD are immune mediated; antibiotics, even if effective in neutralizing or killing bacteria, may not immediately alleviate long-duration immunologic tissue manifestations of antigen stimulation. Conversely, in the absence of the immunologic capability to react to bacterial infection by forming granulomas, as in the case of severely immunocompromised persons with BA, antibiotics are generally effective in alleviating the symptoms and signs of infection. Does this suggest that possible non-granulomatous manifestations of CSD (for example, neuroretinitis and encephalopathy) should respond well to the appropriate antibiotic therapy?

Although BA has been described in immunocompetent patients (15), the vast majority of BA patients are immunocompromised (14). What are the factors explaining why *B. henselae* and *B. quintana* induce vascular proliferative lesions, such as BA and parenchymal bacillary peliosis, almost exclusively in severely immunocompromised patients?

What percentage of the relatively large numbers of undiagnosed febrile disease among HIV-infected persons is in fact due to *Bartonella* species infections? The answer to this important question may help alleviate significant morbidity among HIV-infected patients. The potential for selection for drug-resistance during long-term antimicrobial therapy deserves scrutiny.

Does the 4%-6% of IFA antibody-positive, ostensibly healthy "control" study populations suggest a relatively common undercurrent of undiagnosed, subclinical *Bartonella*-associated disease?

Is it possible to immunize cats and thereby interrupt *B. henselae* transmission to humans? Preliminary data suggest that asymptomatic bacteremia in cats can be successfully treated with antimicrobial therapy (36). Once cleared of bacteremia, are these cats routinely susceptible to reinfection?

Are the complications occasionally associated with CSD and BA associated with different strains of *Bartonella* species or are the variations in clinical presentation strictly functions of dose, route of inoculation, and immune status?

And finally, in what role, if any, will *A. felis* reappear as an agent of human disease? Is *A. felis* responsible for the relatively small number of cases of CSD-like lymphadenopathy that have no evidence of antibody to *B. henselae*? Or is there another explanation for the originally proposed association between *A. felis* and CSD that has not yet come to light?

The new recognition of the importance of *Bartonella*-associated diseases will continue to spawn a host of unanswered related questions. Whereas novel subplots will continue to unfold, the new puzzles are no longer totally shapeless, and answers to questions of natural history and epidemiology, enhanced diagnosis and treatment, and methods for disease intervention should now begin to unfold rapidly.

### References

1. Emmons RW. Cat scratch disease: the mystery finally solved? Ann Intern Med 1984;100:303-4.
2. Goldsmith MF. Has AFIP debugged the cat scratch mystery? JAMA 1983;250:2745-7.
3. Emmons RW, Riggs JL, Schachter J. Continuing the search for the etiology of cat scratch disease. J Clin Micro Biol 1976;4:112-4.
4. Carithers HA. Cat scratch disease: An overview based on a study of 1,200 patients. Am J Dis Child 1985; 139:1124-33.
5. Margileth AM. Cat scratch disease. Adv Pediatr Infect Dis 1993;8:1-21.
6. Karim AA, Cockerell CJ, Petri WA. Cat scratch disease, bacillary angiomatosis, and other infections due to *Rochalimaea*. N Engl J Med 1994;330:1509-15.
7. Zangwill KM, Hamilton DH, Perkins BA, et al. Epidemiology, risk factors, and evaluation of a new diagnostic test. N Engl J Med 1993;329:8-13.
8. Warwick WJ. The cat-scratch syndrome, many diseases or one disease? Prog Med Virol 1967;9:256-301.
9. Johnson WT, Helwig EB. Cat-scratch disease (histopathologic changes in the skin). Arch Dermatol 1969;100:148-54.
10. Wear DJ, Margileth AM, Hadfield TL, Fisher GW, Schlagel CJ, King FM. Cat scratch disease: a bacterial infection. Science 1983;221:1403-5.
11. English CK, Wear DJ, Margileth AM, Lissner CR, Walsh GP. Cat scratch disease: isolation and culture of the bacterial agent. JAMA 1988;259:1347-52.
12. Brenner DJ, Hollis DG, Moss CW, English CK, et al. Proposal to *Afipia* gen. nov., with *Afipia felis* sp. nov. (Formerly the Cat Scratch Bacillus), *Afipia clevelandenis* sp. nov. (Formerly the Cleveland Clinic Strain), *Afipia broomeae* sp. nov., and three unnamed genospecies. J Clin Micro 1991;29:2450-60.
13. Stoler MH, Bonfiglio TA, Steigbigel RT, Pereira M. An atypical subcutaneous infection associated with acquired immune deficiency syndrome. Am J Clin Pathol 1983;80:714-8.
14. Koehler JE, Tappero JW. AIDS Commentary: bacillary angiomatosis and bacillary peliosis in patients infected with human immunodeficiency virus. Clin Infect Dis 1993;17:612-24.
15. Tappero JW, Koehler JE, Berger TG, Cockerell CJ, Lee T-H, Busch MP, Stites DP, Mohle-Boetani J, Reingold AL, LeBoit PE. Bacillary angiomatosis and bacillary splenitis in immunocompetent adults. Ann Intern Med 1993;118:363-5.
16. Perkocha LA, Geaghan SM, Yen TS, et al. Clinical and pathological features of bacillary peliosis hepatis in association with human immunodeficiency virus infection. N Engl J Med 1990;323:1581-6.
17. Kemper CA, Lombard CM, Dersinski SC, Tompkins LS. Visceral bacillary epithelioid angiomatosis: possible manifestations of disseminated cat scratch disease in the immunocompromised host: a report of two cases. Am J Med 1990;89:216-22.
18. Slater LN, Welch DF, Hensel D, Coody DW. A new recognized fastidious gram-negative pathogen as a cause of fever and bacteremia. N Engl J Med 1990;323:1587-93.
19. Black JR, Herrington DA, Hadfield TL, Wear DJ, Margileth AM, Shigekawa B. Life-threatening cat scratch disease in an immunocompromised host. Arch Intern Med 1986;146:394-6.
20. Koehler JE, LeBoit PE, Egbert BM, Berger TG. Cutaneous vascular lesions and disseminated cat scratch disease in patients with the acquired immunodeficiency syndrome (AIDS) and AIDS-related complex. Ann Intern Med 1988;109:449-55.
21. LeBoit PE, Berger TG, Egbert BM, Beckstead JH, Yen TS, Stoler MH. Epithelioid haemangioma-like vascular proliferation in AIDS: manifestation of cat-scratch disease bacillus infection? Lancet 1988;1:960-3.
22. Tappero JW, Mohle-Boetani J, Koehler JE, Swaminathan B, Berger TG, LeBoit PE, Smith LL, Wenger JD, Pinner RW, Kemper CA, Reingold AL. The epidemiology of bacillary angiomatosis and bacillary peliosis. JAMA 1993;269:770-5.

23. Tappero JW, Koehler JE. Cat scratch disease and bacillary angiomatosis [letter]. JAMA 1991;266:1938-39.
24. Relman DA, Loutit JS, Schmidt TM, Falkow S, Tompkins LS. The agent of bacillary angiomatosis: an approach to the identification of uncultured pathogens. N Engl J Med 1990;323:1573-80.
25. Regnery RL, Anderson BE, Clarridge III, JE, Rodriguez-Barradas MC, Jones DC, Carr JH. Characterization of a novel *Rochalimaea* species, *R. henselae*, sp. nov., isolated from blood of a febrile, human immunodeficiency virus-positive patient. J Clin Microbiol 1992;30:265-74.
26. Welch DF, Pickett DA, Slater LN, Steigerwalt AG, Brenner DJ. *Rochalimaea henselae*, sp. nov., a cause of septicemia, bacillary angiomatosis, and parenchymal bacillary peliosis. J Clin Microbiol 1992;30:275-80.
27. Koehler JE, Quinn FD, Berger TG, LeBoit PE, Tappero JW. Isolation of *Rochalimaea* species from cutaneous and osseous lesions of bacillary angiomatosis. N Engl J Med 1992;327:1625-31.
28. Tappero J, Regnery R, Koehler J, Olson J. Detection of serologic response to *Rochalimaea henselae* in patients with bacillary angiomatosis (BA) by immunofluorescent antibody (IFA) testing. Program Abstr. 32nd Interscience Conference on Antimicrobial Agents and Chemotherapy, Anaheim, Calif. Oct 10-14, 1992, Abstr. no. 674.
29. Regnery RL, Olson JG, Perkins BA, Bibb W. Serological response to "*Rochalimaea henselae*" antigen in suspected cat scratch disease. Lancet 1992;339:1443-5.
30. Dolan MJ, Wong MT, Regnery RL, Jorgensen JH, Garcia M, Peters J, Drehner D. Syndrome of *Rochalimaea henselae* adenitis suggesting cat scratch disease. Ann Intern Med 1993;118:331-6.
31. Anderson B, Sims K, Regnery R, Robinson L, Schmidt MJ, Goral S, Hager C, Edwards K. Detection of *Rochalimaea henselae* DNA in specimens from cat scratch disease patients by PCR. J Clin Microbiol 1994;32:942-8.
32. Szelc Kelly C, Edwards KM, Perez-Perez G, Regnery RL, Perkins BA. A new controversy in the etiology of cat scratch disease: *Afipia felis* or *Rochalimaea henselae*? Program Abstr. 32nd Interscience Conference on Antimicrobial Agents and Chemotherapy, Anaheim, Calif., Oct 10-14, 1992. Abstr. no. 1565.
33. Perkins BA, Swaminathan B, Jackson LA, Brenner DJ, Wenger JD, Regnery RL. Case 22-1992—pathogenesis of cat scratch disease [letter]. N Engl J Med 1992;327:1599-600.
34. Anderson B, Kelley C, Threlkel R, Edwards K. Detection of *Rochalimaea henselae* in cat scratch disease skin test antigens. J Infect Dis 1993;168:1034-6.
35. Regnery R, Martin M, Olson J. Naturally occurring "*Rochalimaea henselae*" infection in domestic cat. Lancet. 1992;340:557-8.
36. Koehler JE, Glaser CA, Tappero JW. *Rochalimaea henselae* infection: a new zoonosis with the domestic cat as reservoir. JAMA 1994;271:531-5.
37. Strong RP (ed.) Trench fever: Report of Commission, Medical Research Committee, American Red Cross. Oxford: Oxford University Press, 1918:40-60.
38. Schultz MG. A history of bartonellosis (Carrión's disease). Am J Trop Med Hyg 1968;17:503-15.
39. Lucey D, Dolan MJ, Moss CW, Garcia M, Hollis DG, Wegner S, Morgan G, Almeida R, Leong D, Greisen KS, Welch DF, Slater LN. Relapsing illness due to *Rochalimaea henselae* in immunocompetent hosts: Implication for therapy and new epidemiological associations. Clin Infect Dis 1992;14:683-8.
40. Brenner DJ, O'Connor SP, Winkler HH, Steigerwalt AG. Proposals to unify the genera *Bartonella* and *Rochalimaea*, with descriptions of *Bartonella quintana* comb. nov., *Bartonella vinsonii* comb. nov., *Bartonella henselae* comb. nov., and *Bartonella elizabethae* comb. nov., and to remove the family *Bartonellaceae* from the order *Rickettsiales*. Int J Syst Bacteriol 1993;43:777-86.
41. Noguchi H, Battistini TS. Etiology of Oroya fever. I. Cultivation of *Bartonella bacilliformis*. J Exp Med 1926;43:851-64.
42. Hadfield TL, Warren R, Kass M, Brun E, Levy C. Endocarditis caused by *Rochalimaea henselae*. Human Pathol 1993;24:1140-41.
43. Spach DH, Callis KP, Paauw DS, Houze YB, Schoenknecht FD, Welch DF, Rosen H, Brenner DJ. Endocarditis caused by *Rochalimaea quintana* in a patient infected with human immunodeficiency virus. J Clin Microbiol 1993;31:692-4.
44. Spach DH, Larson AM, Coyle MB, Kanter AS, Welch DF, Stamm AM. Unanticipated *Rochalimaea quintana* bacteremia in patients with chronic alcoholism. [Late Breaker Abstracts]. In: Program Supplement of the 33rd Interscience Conference on Antimicrobial Agents and Chemotherapy. Washington, D.C.: American Society for Microbiology, 1993.
45. Daly JS, Worthington MG, Brenner DJ, Moss CW, Hollis DG, Weyant RS, Steigerwalt AG, Weaver RE, Daneshvar MI, O'Connor SP. *Rochalimaea elizabethae* sp. nov. isolated from a patient with endocarditis. J Clin Microbiol 1993;31:872-81.
46. Myers WF, Grossman DM, Wisseman CL. Antibiotic suseptability patterns in *Rochalimaea quintana*, the agent of trench fever. Antimicrob Agents Chemother. 1984; 25:690-3.
